# Using heterozygosity–fitness correlations to study inbreeding depression in an isolated population of white-tailed deer founded by few individuals

**DOI:** 10.1002/ece3.1362

**Published:** 2014-12-24

**Authors:** Jon E Brommer, Jaana Kekkonen, Mikael Wikström

**Affiliations:** 1 Department of Biology, University Hill20014 University of Turku, Turku, Finland; 2 ARONIA Research and Development Institute at NOVIA University of Applied Sciences and Åbo Akademi UniversityRaseborgsvägen 9, 10600, Ekenäs, Finland; 3 Department of Biosciences, University of HelsinkiPO Box 65(Viikinkaari 1), 00014, Helsinki, Finland; 4 Finnish Wildlife AgencyFantsintie 13-14, 00890, Helsinki, Finland

**Keywords:** Cervid, heterozygosity, inbreeding, introduced population, microsatellite, population genetics

## Abstract

A heterozygosity–fitness correlations (HFCs) may reflect inbreeding depression, but the extent to which they do so is debated. HFCs are particularly likely to occur after demographic disturbances such as population bottleneck or admixture. We here study HFC in an introduced and isolated ungulate population of white-tailed deer *Odocoileus virginianus* in Finland founded in 1934 by four individuals. A total of 422 ≥ 1-year-old white-tailed deer were collected in the 2012 hunting season in southern Finland and genotyped for 14 microsatellite loci. We find significant identity disequilibrium as estimated by *g*_2_. Heterozygosity was positively associated with size- and age-corrected body mass, but not with jaw size or (in males) antler score. Because of the relatively high identity disequilibrium, heterozygosity of the marker panel explained 51% of variation in inbreeding. Inbreeding explained approximately 4% of the variation in body mass and is thus a minor, although significant source of variation in body mass in this population. The study of HFC is attractive for game- and conservation-oriented wildlife management because it presents an affordable and readily used approach for genetic monitoring that allowing identification of fitness costs associated with genetic substructuring in what may seem like a homogeneous population.

## Introduction

Management of populations whose numbers is or has been low requires that genetic aspects are taken on board. In general, low effective population size leads to an increase in inbreeding with an associated depletion of genetic variance and may cause a reduction in population fitness in case of inbreeding depression (Charlesworth and Charlesworth 1987; Crnokrak and Roff 1999). Conservation biologists, for example, need to be concerned when animals are translocated (e.g., a re-introduction or assisted colonization, IUCN 2012), because this procedure typically involves a limited number of individuals with possibly negative impact on the genetic diversity of the founded population (Franklin 1980). Also game managers are recommended to uphold genetic monitoring, because intense management aimed to keep the population far below its carrying capacity is likely to have genetic consequences (Allendorf et al. 2008). In particular, harvesting a population risks lowering the amount of genetic variance (e.g., Scribner et al. 1985; Hartl et al. 1991; Martinez et al. 2002). Whatever causes the altered demography of a population, genetic consequences arise when the population moves away from adhering to the theoretical assumption of being large and panmictic toward having some level of structure. Inbreeding, for example, after a bottleneck or a founding event, is probably the strongest process generating genetic groups within a population (individuals resulting from consanguineous vs. nonconsanguineous matings) (Slate et al. 2004; David et al. 2007; Szulkin et al. 2010; Kardos et al. 2014). Inbreeding depression occurs when inbred individuals perform poorer than outbred ones and knowledge of whether this phenomenon occurs and what its strength is has important ramifications for managing populations (Keller and Waller 2002).

A potentially attractive tool for genetic monitoring is to consider whether variation in individuals' heterozygosity, measured using a set of presumably neutral genetic markers, is associated with a performance trait (Mitton 1993). When present, such heterozygosity–fitness correlation (HFC) implies that low heterozygous individual have a reduced performance, suggesting the occurrence of inbreeding depression. A critique of interpretating HFC as a sign of inbreeding depression is that heterozygosity in the (often limited) set of markers considered need not represent genomewide heterozygosity (general effect hypothesis; Hansson and Westerberg 2002). Many authors have criticized the assumption that a low number of markers would proxy heterozygosity (Balloux et al. 2004; Väli et al. 2008; Ljungqvist et al. 2010; Santure et al. 2010; Taylor et al. 2010; Forstmeier et al. 2012; Kardos et al. 2014; Miller et al. 2014). Alternatively, HFC may arise when one or more of the presumably neutral markers used to quantitate heterozygosity is in linkage disequilibrium with a fitness locus (local effects; Hansson and Westerberg 2002). Typically, HFC has a low effect size (*r *≤* *0.06; Coltman and Slate 2003; Chapman et al. 2009; Miller and Coltman 2014). However, Szulkin et al. (2010) point out that HFC arises as the product of two distinct processes: (1) the degree in which the set of markers reflects genomewide heterozygosity and (2) the degree of inbreeding depression in the population. The former is largely determined by the amount of identity disequilibrium (correlation in heterozygosity across loci in the genome). When identity disequilibrium is low, a typical panel of markers (approximately 10; Miller and Coltman 2014) will poorly reflect genomewide heterozygosity, thereby potentially masking even strong inbreeding depression when only HFC is explored. A recent meta-analysis indeed underlines that identity linkage in most populations studied to date is low: Only 20% (26/129) of estimates show statistically significant identity disequilibrium (Miller and Coltman 2014) as estimated using the *g*_2_ statistic (David et al. 2007). Low identity disequilibrium is found in populations with low variance in inbreeding, which is likely to be common in free-ranging populations (Slate et al. 2004; Kardos et al. 2014). As a consequence, the low effect sizes of most of the available HFC estimates largely reflect the low identity disequilibrium in the populations studied, with the majority of studies on HFC to date being underpowered in terms of the number of markers used to quantitate heterozygosity as a proper correlate of inbreeding (Miller and Coltman 2014). High-throughput sequencing allows obtaining large numbers of markers relatively easy (e.g., Hoffman et al. 2014). Nevertheless, the costs and logistics of obtaining a large number of markers are potentially prohibitive for many end users with conservation or game management interests.

**Figure 1:**
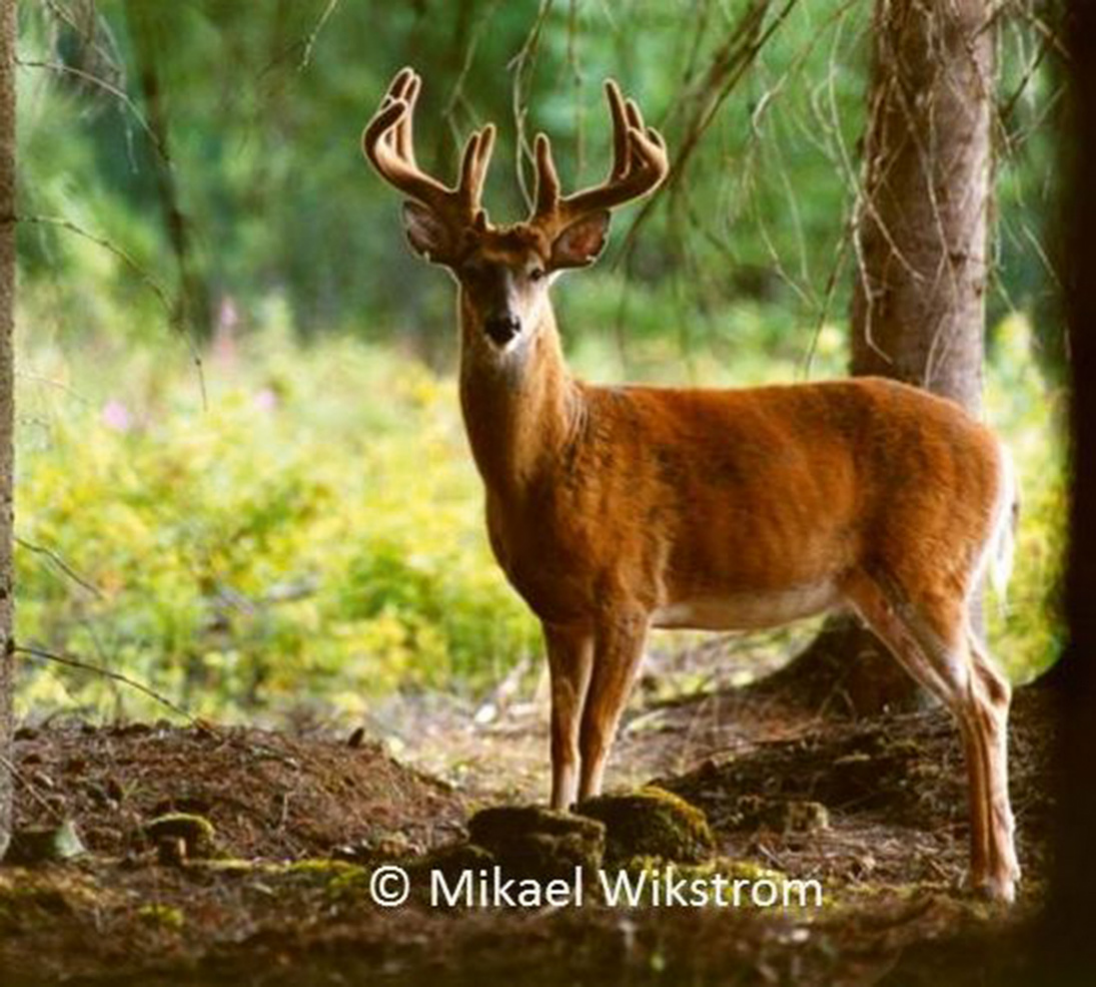
A White-tailed deer male.

In this study, we focus on a population of a large vertebrate with a demographic history that makes it a prime candidate for detecting HFC and identity disequilibrium. We study a white-tailed deer *Odocoileus virginianus* (Fig.1) population in southern Finland. The white-tailed deer's native range is in the Americas, but four individuals of the species were introduced in Finland in 1934 (approximately 26 generations before this study was conducted) with a restocking event in 1948 which may or may not have succeeded (Nygrén 1984; Kekkonen et al. 2012). The population has since rapidly increased in numbers. Because of this numerical increase, the Finnish white-tailed deer has retained high heterozygosity, although it has suffered a reduction in allelic richness (Kekkonen et al. 2012). The Finnish white-tailed deer is, however, an isolated population. White-tailed deer introductions in other European countries have largely failed (Halls 1984), and there is, as far as we are aware, no gene flow between the Finnish population and any other population. During the establishing phase, white-tailed deer have also been translocated repeatedly by moving small numbers of individuals from its national core to other regions of Finland, thereby generating additional founder effects. Harvesting of white-tailed deer started in 1960 (Kekkonen et al. 2012). Currently, the white-tailed deer population in Finland is about 50,000 individuals of which approximately half are harvested annually.

Because of its demographic history, including a substantial bottleneck through founding and translocations, variance in inbreeding is expected to be considerable. We hence anticipate this population to harbor substantial identity disequilibrium. This demographic history therefore creates favorable conditions for finding an association between heterozygosity and performance traits, provided there is inbreeding depression (Szulkin et al. 2010; Kardos et al. 2014). Ideally, HFCs are explored for estimates of individual fitness (Slate et al. 2000; Da Silva et al. 2009). Nevertheless, HFCs are also commonly performed on morphological or growth traits under the assumption of these correlate to fitness (Miller and Coltman 2014). We here consider the aspects of an individual's size, body mass after slaughter, and skeletal size (dimensions of the lower jaw) as measures of individual performance, as well as the size of antlers in males. Cervids are sexually dimorphic and increase in size during the first year of their life (Brown 1992). We therefore corrected body mass, jaw size, and antler size for variation across age and sex. Our general hypothesis is that inbreeding produces individuals, which are relatively small compared to others in their age and sex group, thereby reflecting poorer growth of inbred white-tailed deer (cf. Leberg et al. 1992). In particular, body mass is considered a fitness-related trait in white-tailed deer. Heavier white-tailed deer females are more fecund (Cothran et al. 1987), and heavier white-tailed deer males have a larger mating success (Jones et al. 2011). Furthermore, an association between heterozygosity and weaponry is found in cervids and sheep including white-tailed deer (e.g., Scribner and Smith 1990; Hartl et al. 1995; Ditchkoff et al. 2001; von Hardenberg et al. 2007; Pérez-González et al. 2010), suggesting that antlers are prime candidates for detecting inbreeding depression.

## Materials and Methods

### Material collected

Volunteer hunters provided heads from ≥1-year-old individuals shot during the hunting season September 2012 – January 2013. In Finland, white-tailed deer hunting is based on quota determined by governmental authorities for each region. Quota specifies the number of calves, females, and males which can be shot by a hunting group, but there are no legal restrictions on sizes or other characteristics for the harvested animals. Hunters did not receive monetary or other compensation for providing samples, nor were they otherwise influenced to adjust their hunting effort. The study area covered the municipalities of Hanko, Raasepori, and Inkoo (central coordinate 60°3′N, 23°18′E; approximately 140,000 ha), which is an area with abundant white-tailed deer. Overall, 70 hunting groups were involved in collecting the material for this study. Although certain hunting groups may adhere to a voluntary agreement for selective hunting, we believe that the combined material provides a decent representation of the variation in size traits within each sex and age group. Approximately 90% of all adult individuals shot in this area during the 2012–2013 hunting season were included in this study, which thereby provides a representative sample of a white-tailed deer population.

After the intestines, head, skin, and hooves were removed, body mass after slaughter was measured in kilograms using a spring scale by the hunters who harvested the animal. Lower jaws were removed, skinned boiled, and cleaned. Using a sliding caliper, author JK measured six dimensions of the jaw. (1) Jaw length: distance from the most lateral point of the alveolar margin of the canine socket to the posterior border of the angle (0.5 mm accuracy); (2) Height of the mandibular notch: distance from the most ventral point of the notch to the most ventral point of the ventral border of the angle (0.1 mm accuracy); (3) Mandibular body height: the minimum distance from the dorsal border of the body medial to the anterior cusps of the first molar to the ventral border of the body (0.1 mm accuracy); (4) Diastema length: distance from the most lateral point of the alveolar margin of the canine socket to the most anterior point of the alveolar margin of the P_2_ socket (0.1 mm accuracy); 5) Diastema height: the minimum distance between the dorsal and ventral borders of the diastema posterior to the mandibular symphysis (0.1 mm accuracy); and 6) Diastema width: the minimum mediolateral distance through the diastema posterior to the mandibular symphysis (0.1 mm accuracy). These measures (described in Appendix 1) are comparable to North American studies (Rees 1969). The first principal component in a principal component analysis (PCA) of all jaw measures (Appendix 2) was extracted and used as the metric for jaw size. For some individual, one or more of aspects of the size of the jaw could not be measured due to damage to the bone and these values were replaced by their sex- and age-specific mean value in the PCA. Because this is a principal component, the unit is standard deviation.

The size of antlers was quantitated as its score following the system of the International Council for Game and Wildlife Conservation (CIC). The CIC antler score was chosen because it is a composite of a large number of features of the antlers and size measures, including length of the main beam, brow tine, and tray tine, circumference of the lower and upper beams (all measured on left- and right-hand sides), number of points, and span of antlers. Scoring was performed by CIC-certified measurers. A larger CIC antler score reflect an overall more voluminous pair of antlers.

One-year-old white-tailed deer can be recognized reliably from dental patterns, because their third premolar teeth are not changed yet and has three cusps instead of the two as found in individuals aged 2 years or older. For individuals older than 1 year, an incisor tooth of the lower jaw (with roots) was aged by Matson's laboratory (URL http://www.matsonslab.com/for-private-hunter-outfitter-and-hunting-club-clients.html), which is a certified laboratory for aging large game animals, based on their protocol for highest accuracy cementum aging.

### Analyses of genotype data

DNA was extracted from a small piece of muscle following the method described in Elphinstone et al. (2003), except that 70 *μ*L of dH_2_O was used to elute DNA in the last step. The samples were amplified in polymerase chain reaction (PCR) for fourteen microsatellite loci in four parallel panels. PCR was conducted with Phusion Flash master mix (Finnzymes), where one PCR contained 5 *μ*L of master mix solution, 2 *μ*L of extracted DNA, 1 *μ*L of dH_2_O, and 2 *μ*L of primer mix. The final concentration of all primers was 0.5 *μ*mol/L. Panel 1 included primers INRA011 (Vaiman et al. 1992) and Cervid1 (DeWoody et al. 1995). Panel 2 included N, Q (Jones et al. 2000), ETH152 (Steffen et al. 1993), and BM203 (Bishop et al. 1994). Panel 3 included K (Jones et al. 2000), BL25, BM6438, BM848 (Bishop et al. 1994), and O (Jones et al. 2000). Panel 4 included BM6506, (Bishop et al. 1994), D (Jones et al. 2000), and OarFCB193 (Buchanan and Crawford 1993). PCRs for all panels were completed on a Bio-Rad S1000 thermal cycler, using the following protocol (annealing temperature 58°C for panels 1 and 2 and 54°C for panels 3 and 4): one denaturing step of 10 s at 98°C followed by 30 cycles of 1s at 98°C, 5s at 58°C or 54°C depending on the panel, and 15s at 72°C. Finally, there was an additional 1 min at 72°C and an indefinite hold at 4°C. Forward primers were fluorescently labeled with FAM, HEX or TAMRA labels and PCR products were separated and visualized with an ABI 3730 sequencer (Applied Biosystems, Waltham, MA, USA). Genotypes were scored using the software package Genemapper vs 4.1 (Applied Biosystems). The loci were checked for Hardy–Weinberg equilibrium with software Cervus (Kalinowski et al. 2007) and for linkage disequilibrium with software Genepop 3.4 (Raymond and Rousset 1995). Some samples were re-extracted due to weak products (12 samples). Regenotyping was performed to samples for which not all genotypes could be read from the first run. When regenotyping, the whole panel was run again which provided a control at the same time. Moreover, some samples which had all loci successfully genotyped were rerun for control. Altogether, regenotyping was performed so that the genotypes could be checked for 9% of the samples, and the agreement was excellent.

Program INEst 1.0 (Chybicki and Burczyk 2008) was used to estimate inbreeding and null allele frequencies in the loci of 241 individuals for which all 14 loci were successfully amplified. Population Inbreeding model (Standard EM estimation) accounting for inbreeding and genotype failures was used.

### Analyses of heterozygosity–fitness correlations and identity linkage

We followed the guidelines detailed in Szulkin et al. (2010). Multilocus heterozygosity of individual *i* (*H*_*i*_) was calculated as

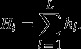
1where for each of *L* loci, *h*_*l*_ scores whether the *l*-th locus is heterozygous (1) or not (0). If that locus failed to amplify for individual *i,* the average heterozygosity of the locus over all individuals for which it was available was used instead. Identity disequilibrium was estimated using the *g*_2_ parameter for the 14 loci with the program RMES (David et al. 2007), which takes into account missing values at loci. RMES provides a test of significance under the null hypothesis *g*_2_ = 0 based on 1000 permutations.

We tested the effect of *H* on the focal trait, in a model where the trait was always corrected for the age and (for body mass and jaw length) the sex of the individual including the interaction age × sex (when relevant). Age was categorized into five classes (1, 2, 3, 4, and 5+). Age and sex differences were accounted for because male white-tailed deer grow in size during their first 4 years, whereas females typically change little in size after their second year (Leberg et al. 1992). Linear models including the main effect of *H* and those additionally including interactions of the explanatory variables with *H* were computed and ranked on the basis of their Akaike information criterion AIC (Burnham and Anderson 2002). We calculated ΔAIC as the difference in AIC score between the top model (lowest AIC) and each candidate model. Akaike weights were calculated as exp (-½ ΔAIC)/Σ(exp (-½ ΔAIC)) over all candidates models; these indicate relative support for a particular model in comparison to the other candidate models (Burnham and Anderson 2002).

We focused on the main effect of *H* on our focal traits *T* defined as the residuals after correcting for age and sex differences and their interaction. Note that this procedure does not change the scale of the original data. We then estimated

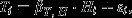
2

which is a linear regression equation where *β*_*T, H*_ denotes the fixed-effect slope of multilocus heterozygosity *H* on the trait value *T* and *ε* denotes the residual. The local effect hypothesis was tested following the guidelines of Szulkin et al. (2010), where the model of eq.2 was compared using ANOVA with a model with the *h* value (eq. 1) for all fourteen microsatellite loci with any missing values replaced by their average. When this test shows a nonsignificant difference, the most parsimonious model (eq. 2) is preferred. The coefficient of determination *r*^2^_*T*, *H*_ of the model specified in eq. 2 was used to calculate the coefficient of determination for the effect of the inbreeding coefficient *f* on trait *T* as detailed by the formulas derived by Szulkin et al. (2010).

### Data included

Not all aspects of morphology and genotypes were available for all individuals. For some (18) individuals, DNA amplification failed completely, probably because of sample quality deterioration due to delayed taking of the DNA sample after the individual was harvested. We included DNA samples of individuals for which morphometrics was not available (e.g., traffic accidents). Body mass after slaughter was not always provided by the volunteer hunters. Some of the antlers were damaged during the hunt or transport and could not be scored. Thus, HFC and identity disequilibrium as well as calculations for inbreeding depression were conducted on data sets specific to each trait, as detailed in the Results. As in all studies conducted on wild animals, our study suffers from ignorance of the “invisible fraction” (Grafen 1988) of individuals that died before being measured. In particular, we here included 1-year-old and older individuals and any decrease in performance due to inbreeding during the first year of life hence remains hidden from our analysis. We analyzed whether viability selection on *H* occurred in the cross-sectional data by testing whether *H* differed between the age groups.

### Power analysis

We calculated the power as a function of the number of microsatellites for the size trait for which we detected a significant HFC (see Results). *H* was calculated for 5, 7, 9, 11, or 13 of the 14 microsatellites available. The required number of microsatellites was randomly drawn, and a linear model was performed including age, sex, and their interaction in addition to the *H* based on the randomly drawn set of microsatellites. The procedure was repeated 10,000 times, and power was calculated as the fraction of these times a significant (*P* < 0.05) effect of *H* was detected.

### Implementation of analyses

All analyses, except the calculation of identity disequilibrium, were conducted in R (R Core Team 2014). Script for the analyses is provided in the Supplement (Text S1).

## Results

### Genetic diversity

On average, 88.5% of all individuals were typed per locus (as estimated by CERVUS). Thirteen of fourteen loci were found to be in Hardy–Weinberg equilibrium. The locus that was not in equilibrium was BM203 (*P* = 0.0002). Only one pair of loci showed weak evidence of linkage disequilibrium (loci INRA011 and Q), which was significant after Bonferroni correction for multiple comparisons (*P* < 0.0006). Eleven of fourteen of the loci had null allele frequencies of zero or <0.01 (Table1). In one case, the frequency was over 0.05 (BM203). There was no population-wide inbreeding detected (*F*_IS_ < 0.0001). We found a high level of genetic diversity (Table1), similar to our previous white-tailed deer work based on 72 individuals from the same area (Kekkonen et al. 2012). On average, more than 9 of the 14 loci were heterozygous in an individual (Table1). Identity linkage as estimated by the *g*_2_ (Table1) was statistically different from zero. Heterozygosity did not changes across the age groups (*F*_4,389_ = 1.94, *P* = 0.10), suggesting that older individuals were not a subset of ≥1-year-old individuals selected for higher heterozygosity.

**Table 1:** Microsatellite loci are used in this study. Their allelic richness (AR) and average heterozygosity (E(*h)*. eq. 1) of each locus as calculated over the *N* individuals for which the locus amplified successfully, except that the sample size for the calculation of null alleles was 241. Provided are the summary statistics including the average multilocus heterozygosity 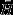 and its variance *σ*^2^(*H*) as well as *g*_2_ with standard deviation (SD) as a measure for identity linkage. A total of 422 individuals were genotyped and the summary statistics are for this set of individuals where missing locus-specific values were taken into account

Locus	AR	E(*h*)	*N*	Null
BL25	5	0.54	397	0.010
BM203	9	0.64	359	0.078
BM6438	5	0.64	365	0.005
BM6506	5	0.61	384	0.050
BM848	8	0.72	383	0.023
Cervid1	7	0.72	391	0.002
D	8	0.66	388	0.005
ETH152	7	0.81	339	0.000
INRA011	6	0.69	378	0.000
K	3	0.51	379	0.000
*N*	11	0.77	344	0.000
O	4	0.56	409	0.000
OarFCB193	7	0.77	358	0.000
Q	9	0.76	360	0.000
Statistic			Value	
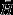			9.385	
*σ*^2^(*H*)			2.802	
*g*_2_ ± SD			0.01624 ± 0.004638	

### Body mass, jaw size, and antler score in relation to age and heterozygosity

The descriptive statistics of the size traits for the individuals included in the HFC analysis (Appendix 1) revealed considerable variation. Individual heterozygosity positively correlated with age- and sex-corrected body mass, and omitting heterozygosity from the model deteriorated model fit by 3.41 AIC (Table2). Inclusion of interaction terms involving heterozygosity did not improve model fit (Table2). Heterozygosity did not have an effect on jaw size or antler score (Table2).

**Table 2:** AIC scores for models for the effect of heterozygosity (*H*) and its interactions with age (in 5 classes) and sex on body mass, jaw size, and CIC antler score. White-tailed deer increase in size during the first 5 years of life and are sexually dimorphic, and hence, all models on body mass and jaw size included age classes, sex, and their interaction, and all models on antler score corrected for age differences. Omission of any of these effects caused a strong rise in AIC. Analysis of body mass was based on 160 females and 151 males, for jaw size on 206 females and 187 males and for CIC antler score on 164 males. Notation of models was such that an interaction was shorthand for also including all lower order interactions and main effects of the variables included in the interaction (e.g., “Age × Sex” stands for “Age + Sex + Age × Sex”). The most parsimonious model is printed in bold

Trait	Model	AIC	ΔAIC	AIC weight
Body mass	**Age × Sex + *****H***	2197.58	0	0.48
	Age × Sex + *H *× Sex	2199.45	1.87	0.19
	Age × Sex + *H *× Age	2199.73	2.15	0.17
	Age × Sex	2200.99	3.41	0.09
	Age × Sex + *H *× Sex + *H *× Age	2201.73	4.15	0.06
	Age × Sex × *H*	2205.27	7.69	0.01
Jaw size	**Age × Sex**	1194.47	0	0.55
	Age × Sex + *H*	1195.66	1.19	0.31
	Age × Sex + *H *× Sex	1197.66	3.19	0.11
	Age × Sex + *H *× Age	1201.10	6.63	0.02
	Age × Sex + *H *× Sex + *H *× Age	1203.08	8.61	0.00
	Age × Sex × *H*	1206.26	11.79	0.00
Antler score	**Age**	1686.42	0	0.66
	Age + *H*	1687.98	1.56	0.30
	Age + *H *× Age	1692.30	5.88	0.04

**Figure 2:**
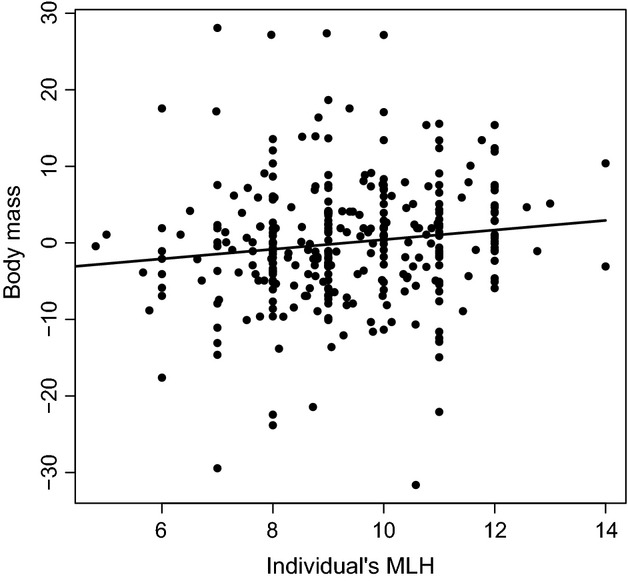
Residual body mass (in kg) against multilocus heterozygosity (sum of heterozygous loci over all 14 loci, eq. 1). Residuals are taken from a regression of body mass after slaughter against age, sex, and their interaction. Line drawn is the regression with the slope and it significance given in Table3.

Although statistically significant, the effect size of heterozygosity on body mass was low, explaining only 1.7% of the variation in age- and sex-corrected body mass (Fig.2; Table3). Exclusion of the putative null allele locus BM203 did not changed the results qualitatively (*β*_*T, H*_ ± SE = 0.60 ± 0.29, *P* = 0.04; 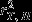 = 1.3%). Body mass increased approximately 6 kg over the observed range in multilocus heterozygosity (Fig.2). We found no evidence supporting the local effect hypothesis for body mass (*F*_13,287_ = 0.54. *P* = 0.9). Based on the estimates of genetic diversity and identity disequilibrium (Table1, Appendix 3), it was clear that there was a reasonable association between marker heterozygosity and inbreeding underlying the heterozygosity – trait correlations, but a weak effect of inbreeding of trait variation (Table3). For comparison, we also show that inbreeding explains an order of a magnitude less variation in age- and sex-corrected jaw size and age-corrected antler score, which were traits that did not have a significant relationship with heterozygosity (Table2).

**Table 3:** Calculation of heterozygosity–fitness correlation and its underlying statistics based on formulas derived by Szulkin et al. (2010). See Table2, for sample sizes. Estimates of 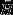, *σ*^2^(*H*) and *g*_2_ for the genotyped individuals considered in each of these three analyses are reported in Appendix 3, but agree well with those presented for all 422 genotyped individuals in Table1

Equation	Value for body mass	Value for jaw size	Value for antler score
Regression slope of trait on heterozygosity			
*β*_*T, H*_ ± SE	0.67 ± 0.28^a^	–0.029 ± 0.033^b^	–1.18 ± 1.83^c^
Proportion of trait variation explained by heterozygosity			
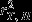	0.0165	0.0021	0.0025
Proportion of variation in inbreeding explained by marker heterozygosity			
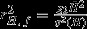	0.42	0.44	0.64
Proportion of trait variation explained by variation in inbreeding			
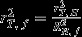	0.039	0.0048	0.0039

^a^*t*_309_^ ^=^ ^2.3, *P* = 0.024; ^b^*t*_391_^ ^=^ ^0.89, *P* = 0.38; ^c^*t*_162_ = 0.64, *P* = 0.052.

The power for detecting a significant relationship between heterozygosity and body mass declined rapidly with decreasing number of markers and was 0.145, 0.228, 0.371, 0.571, and 0.763 for 5, 7, 9, 11, and 13 loci, respectively.

## Discussion

Four white-tailed deer (three males and one female) were introduced in Finland from northern America in 1934 with a possible restocking by an additional four individuals (one male and three females) in 1948 (Nygrén 1984). The population has remained isolated as it was founded. When a population originates from such few individuals, variance in inbreeding is expected to be substantial, which creates a form of within-population structuring establishing identity disequilibrium (Szulkin et al. 2010). We indeed find that the *g*_2_ metric, which is a measure of identity disequilibrium (David et al. 2007), is significant. Miller and Coltman (2014) found that published studies had an average *g*_2_ of 0.007 with only 26 of 129 estimates of *g*_2_ significantly different from zero. The Finnish white-tailed deer's *g*_2_ (0.016) is about double this average and falls in the top 10% of available *g*_2_ estimates (Miller and Coltman 2014). Thus, we find substantial identity disequilibrium. As a consequence, the heterozygosity of fourteen microsatellite loci explained 51% of the variation in inbreeding in this population. That is, the expected correlation between heterozygosity and inbreeding is √0.51 = 0.71 in this population. This is a decently high value. Lastly, we find that despite the significant association between heterozygosity and body mass, inbreeding explains a modest 3.9% of the variation in body mass reflecting low inbreeding depression in body mass. The heterozygosity–fitness correlation (HFC) is the product of these two processes; hence, the heterozygosity of our marker panel explained a low proportion (1.6%) of variation in body mass.

Our findings are encouraging for those interested in genetic monitoring of wild populations. Our study illustrates that when the focal population has undergone a severe bottleneck (e.g., through founding or through temporary low population size), HFC studies based on relatively few markers may be sufficient to provide a good handle on whether inbreeding depression is an important source of variation in the study population. Arguably, populations with such a demographic history are most likely to be of interest, and in need of, genetic monitoring. At the very least, a study with a limited marker set will allow a calculation of the point estimates for identity disequilibrium and the effect of inbreeding on trait variation as exemplified in this study. These estimates will allow making informed decisions on whether a larger set of markers need to be developed and, if so, how many markers would be required (e.g., Miller et al. 2014). In our case, retrospective power analysis underlined that >10 microsatellites are needed to perform analysis with decent power, despite the high identity disequilibrium in this population. As noted by Szulkin et al. (2010) and demonstrated in this study, the traditional population genetics F_IS_ statistic provides a much less sensitive measure of inbreeding in the study population than individual-based approaches. Nevertheless, the notably high heterozygosity and allelic richness of white-tailed deer (Breshears et al. 1988; Anderson et al. 2002) likely facilitated the feasibility of working with a relatively small number of markers in our case.

One restriction in our analysis, and in most HFC studies, is that there may be an “inivisible fraction” (Grafen 1988) of individuals which remained unmeasured. In our case, we did not consider individuals during their first year of life. Severely inbred calves may, for example, suffer increased mortality, and hence, the individuals considered in our sample may represent a selected subset where the effects of inbreeding depression are lower than in the population as a whole. As a consequence, our conclusion on HFC need to be interpreted with respect to the age classes considered.

In contrast to our expectations, we here find no evidence that heterozygosity is associated with the development of antlers in the Finnish white-tailed deer population. One factor which may explain our findings is that male mating success in white-tailed deer is not strongly associated with antler size, but is instead largely determined by the demography (adult sex ratio and age distribution of males) of the population (DeYoung et al. 2006, 2009). Because antler size is a male trait, the heterozygosity–antler size analysis has approximately half the sample size of our analyses of the other traits. Because of this reduction in sample size, analysis of a male-specific trait (such as antler score) will, for a given effect size of HFC, have lower power relative to traits expressed in both sexes. Nevertheless, the estimated effect sizes of the associations between heterozygosity and jaw size, and heterozygosity and antler score are low (*r*^2^ < 0.3%), suggesting that prohibitively large sample sizes would be needed to reach statistical significance. Even for body mass, which showed significant correlation with heterozygosity, the effect size was still small (*r*^2^ = 1.7%). Leberg et al. (1992) studied a large number of white-tailed deer and concluded that the quality of habitat is the most important determinant of growth in free-ranging white-tailed deer individuals. Thus, the generally modest effect of inbreeding on our size measures (although significant for body mass) is hence not surprising.

In conclusion, we here separate the strength of the correlation between the heterozygosity of the marker panel and inbreeding from the effect of inbreeding on the performance trait, as advocated by Szulkin et al. (2010). We show that this approach allows for a powerful first-line investigation into the putative negative consequences of inbreeding in a vertebrate population founded by few individuals. This procedure allows genetic monitoring, of interest for conservation biologists working with populations that are either founded by few individuals or which have been reduced to a small size in the past. In addition, game managers should be concerned about inbreeding as it signals genetic change in a harvested population. Such changes generally make it more difficult to manage populations in a sustainable manner (Ratner and Lande 2001; Proaktor et al. 2007).

## Data Accessibility

Morphological measures and heterozygosity of 14 microsatellite loci of white-tailed deer included in this study. doi: 10.5061/dryad.f6q02.

## Appendix 1

Descriptive statistics of the measures taken. Body mass and antler score were analyzed as separate traits, but the various jaw measures taken were combined in a principal component analysis PCA (See Appendix 2), and PC1 of jaw size was used as a metric in the HFC analyses. Body mass (after slaughter) taken in kg. For antler score, the CIC method was followed which combines a number of metrics into a dimensionless score, where a larger score indicates larger antlers. Sample sizes for body mass, jaw size, and antler score include the number of males and females with measures for these traits as well as genotype data (i.e., data used in Table2 and Table3). The PCA on jaw size included six jaw measures (in mm) taken on all individuals (also including some which were not genotyped), and we here present the sample size and summary statistic for each jaw measure. Some measures could not be taken because of damages to the bone and missing values were replaced by the mean, as reported in this table.

**Table:**

	Male				Female						
Measurement	*N*	Min	Max	Mean	*N*	Min	Max	Mean
Body mass	151	30.0	98.0	54.4	160	14.0	71.5	39.5
Jaw size (PC1)	187	5.4	–2.8	1.2	206	3.6	–4.5	–0.9
Antler score	164	12	383	168.4				
Jaw measure (all individuals)								
Jaw length	209	192.50	250.00	218.19	237	182.50	231.50	208.30
Mandibular notch height	208	59.86	85.82	71.98	227	53.36	79.84	67.35
Mandibular body height	215	19.98	32.84	25.88	245	19.29	27.93	23.35
Diastema length	215	61.35	91.60	74.03	245	54.71	85.17	68.17
Diastema height	214	11.62	20.40	15.89	246	11.13	18.08	14.07
Diastema width	214	5.41	10.29	7.76	246	5.52	9.50	6.98

## Appendix 2

Principal component analysis (PCA) of several aspects of the dimension in jaw size. Descriptive statistics reported in Appendix 1. All measure loaded in the same direction on PC1 which was used in the analyses as a measure of jaw size. In the PCA, missing values were replaced by the age- and sex-specific mean value.

**Table:**

	PC1	PC2	PC3	PC4	PC5	PC6
SD	2.13	0.79	0.57	0.50	0.42	0.26
%Var	75.7	10.6	5.4	4.2	3.0	1.1
Σ %Var	75.7	86.3	91.7	95.9	98.9	100

Jaw dimension	Loadings on PC1
Jaw length	0.43
Mandibular notch height	0.42
Mandibular body height	0.40
Diastema length	0.42
Diastema height	0.38
Diastema width	0.39

## Appendix 3

Parameters for the subsets of all genotyped individuals used in specific analyses reported in Table3 in the main text. The parameter g_2_ was calculated on the basis of the genotypes of the individuals included in the analysis (sample size *N*) in RMES (David et al. 2007) which allows for missing genotype values. Average and variance in multilocus heterozygosity (E(*H*) and var(*H*), respectively) was calculated for the *N* individuals included in each particular analysis where the average locus-specific heterozygosity over all genotyped individuals (Table2) was used to replace any missing locus-specific heterozygosity scores.

**Table:**

Parameter	Body mass	Jaw size	CIC antler score
*N*	311	393	164
g_2_ ± SD	0.013 ± 0.0050	0.014 ± 0.0045	0.022 ± 0.0086
E(*H*)	9.33	9.37	9.23
var(*H*)	2.72	2.77	2.94

## Supporting Information

**Text S1.**R script for running the analyses.
