# Supplementary material for: Using heterozygosity–fitness correlations to study inbreeding depression in an isolated population of white-tailed deer founded by few individuals
Source: Ecol Evol. 2014 Dec 24;5(2):357–67. doi: 10.1002/ece3.1362 (PMC4314268; doi:10.1002/ece3.1362)
Supplement: Supplementary file 1 [file ece30005-0357-sd1.docx]

**Electronic Supplement**

belonging to the article

**Using Heterozygosity-Fitness-Correlations to study inbreeding depression in an isolated population of white-tailed deer founded by few individuals**

by JE Brommer, J Kekkonen and M Wikström

Text S1. R script for the analyses presented in this paper. The script is based on the data file with all measures and microsatellite heterozygosity provided in Dryad.

#¤¤¤¤¤¤¤¤¤¤¤¤¤¤¤¤¤¤¤¤¤¤¤¤¤¤¤¤¤¤¤¤¤¤¤¤¤¤¤¤¤¤¤¤¤¤¤¤¤¤444¤¤¤¤¤¤¤¤¤¤¤¤¤¤¤¤¤¤¤¤¤¤¤¤¤¤¤¤¤

# R script belonging to the article "Using Heterozygote-Fitness-Correlations to

# study inbreeding depression in an isolated population of white-tailed deer

# founded by few individuals by Brommer, Kekkonen, Wikström

#

# This script uses the data deposited in Dryad (DOI:10.5061/dryad.f6q02)

# and implements the analyses reported in the article.

# Its purpose is to clarify and facilitate implementation on other data

# code by Jon E. Brommer, written under R vs. 3.0.0

#¤¤¤¤¤¤¤¤¤¤¤¤¤¤¤¤¤¤¤¤¤¤¤¤¤¤¤¤¤¤¤¤¤¤¤¤¤¤¤¤¤¤¤¤¤¤¤¤¤¤¤¤¤¤¤¤¤¤¤¤¤¤¤¤¤¤¤¤¤¤¤¤¤¤¤¤¤¤¤¤¤¤

wtd.dat<-read.table("WTDdata.txt", header=T)

# above reads the data deposited in Dryad when saved to your working directory

head(wtd.dat) #check it makes sense

#prepare output file for RMES, loci where heterozygosity is missing should be

# changed to have the code for missing value (-99)

loci<-wtd.dat[,13:26]

write.table(ifelse(loci==0,0,ifelse(loci==1,1,-99)),"WTDgenotypes.txt",sep=" ",col.names=F,row.names=F)

# g2 calculated by RMES (David et al. 2007): the output file needs to modified slightly in a text editor

# follow RMES instructions. The first lines are to read

#1

#WTD

#422

#14

#--- followed by the above created output file

#for the above data on 422 individuals it produces

#g2= 0,0162427109435706 ; sd= 0,00463860340965402 ; expected bias = 4,55760862821702E-5

#s(g2)=0,0601450104236704 ; sd= 0,0159148431804892 ; expected bias = -0,000173628402122834

#p-value (H0: g2=s=0) = 0 based on 1000 iterations

#--etc-

#average MLH

mean(wtd.dat$Sum.H,na.rm=T) #[1] 9.385176

#variance in MLH

var(wtd.dat$Sum.H,na.rm=T) #[1] 2.802237

#####################################################################################

## PCA

#¤¤¤¤¤¤¤¤¤¤¤¤¤¤¤¤¤¤¤¤¤¤¤¤¤¤¤¤¤¤¤¤¤¤¤¤¤¤¤¤¤¤¤¤¤¤¤¤¤¤¤¤¤¤¤¤¤¤¤¤¤¤¤¤¤¤¤¤¤¤¤¤¤¤¤¤¤¤¤¤¤¤

replace the missing jaw measures with the age and sex specific means to allow PCA for all individuals

#¤¤¤¤¤¤¤¤¤¤¤¤¤¤¤¤¤¤¤¤¤¤¤¤¤¤¤¤¤¤¤¤¤¤¤¤¤¤¤¤¤¤¤¤¤¤¤¤¤¤¤¤¤¤¤¤¤¤¤¤¤¤¤¤¤¤¤¤¤¤¤¤¤¤¤¤¤¤¤¤¤¤

#morph.means per age and sex

#jawlength

morph.means<-aggregate(wtd.dat$jawlength,list(wtd.dat$age,wtd.dat$Sex),function(m) mean(m,na.rm=T))

names(morph.means)=c("age","sex","jl.mean")

#mnotchheight

mnotch.means<-aggregate(wtd.dat$mnotchheight,list(wtd.dat$age,wtd.dat$Sex),function(m) mean(m,na.rm=T))

names(mnotch.means)=c("age","sex","mnotch.mean")

morph.means<-merge(morph.means,mnotch.means, all.x=T)

#mbodyheight

mbody.means<-aggregate(wtd.dat$mbodyheight,list(wtd.dat$age,wtd.dat$Sex),function(m) mean(m,na.rm=T))

names(mbody.means)=c("age","sex","mbody.mean")

morph.means<-merge(morph.means,mbody.means, all.x=T)

#dlength

dlength.means<-aggregate(wtd.dat$dlength,list(wtd.dat$age,wtd.dat$Sex),function(m) mean(m,na.rm=T))

names(dlength.means)=c("age","sex","dlength.mean")

morph.means<-merge(morph.means,dlength.means, all.x=T)

# dheight

dheight.means<-aggregate(wtd.dat$dheight,list(wtd.dat$age,wtd.dat$Sex),function(m) mean(m,na.rm=T))

names(dheight.means)=c("age","sex","dheight.mean")

morph.means<-merge(morph.means,dheight.means, all.x=T)

# dwidth

dwidth.means<-aggregate(wtd.dat$dwidth,list(wtd.dat$age,wtd.dat$Sex),function(m) mean(m,na.rm=T))

names(dwidth.means)=c("age","sex","dwidth.mean")

morph.means<-merge(morph.means,dwidth.means, all.x=T)

#¤¤¤¤

# replace NA's for the PCA

for (col in 2:7) {

for (i in 1:dim(wtd.dat)[1]) {

if (( is.na(wtd.dat[i,col])) & (sum(is.na(wtd.dat[i,2:7]))<6) ) {

wtd.dat[i,col]<-morph.means[which((morph.means[,1]==wtd.dat$age[i])&(morph.means[,2]==wtd.dat$Sex[i])),(col+1)]

} #if

} # for (i

} # for col

##

wtd.jaw.dat<-subset(wtd.dat, complete.cases(wtd.dat[,2:7]),select=c("Code", "jawlength","mnotchheight","mbodyheight","dlength","dheight","dwidth"))

PCA.jaw<-prcomp(wtd.jaw.dat[,2:7],scale=T)

plot(PCA.jaw)

summary(PCA.jaw)

wtd.jaw.dat$PC1.jaw<-predict(PCA.jaw)[,1]

wtd.dat<-merge(wtd.dat,subset(wtd.jaw.dat,select=c("Code","PC1.jaw")),all.x=T)

##################################################

# analysis

##################################################

#mass

# analysis stepwise

wtd.weight.complete<-subset(wtd.dat,select=c("weight","age.class","Sex","Sum.H"))

wtd.weight.complete<-wtd.weight.complete[complete.cases(wtd.weight.complete),]

#sample size

aggregate(weight~Sex,data=wtd.weight.complete,length)

#effect sizes and test

summary(lm(formula = weight ~ -1 + age.class + Sex + Sum.H + age.class:Sex,data = wtd.weight.complete))$coeff

# effect size

res.weight<-residuals(lm(weight~age.class*Sex, data=wtd.weight.complete))

summary(lm(res.weight~wtd.weight.complete$Sum.H)) #gives the coefficient for the HFC (0.63) and the r^2 (0.0165)

#plotting the data and the regression

plot(res.weight~wtd.weight.complete$Sum.H,pch=19,xlab=list("Individual's H", cex=1.4),ylab=list("Residual body mass", cex=1.4))

H.min.max<-c(4.6,14)

coef.res.weight<-lm(res.weight~wtd.weight.complete$Sum.H)$coef

lines(H.min.max,c(coef.res.weight[1]+ coef.res.weight[2]*H.min.max[1],coef.res.weight[1]+ coef.res.weight[2]*H.min.max[2]),lwd=2.5)

#genotpyes to calculate the g2

genotypes.weight.complete<-subset(wtd.dat,select=c("weight","age.class","Sex","Sum.H","BL25","BM203","BM6438","BM6506","BM848","Cervid1","D","ETH152","INRA011","K","N","O","OarFCB193","Q"))

genotypes.weight.complete<-genotypes.weight.complete[complete.cases(genotypes.weight.complete[,c("weight","age.class","Sex","Sum.H")]),]

genotypes.weight.complete<-subset(genotypes.weight.complete,select=c("BL25","BM203","BM6438","BM6506","BM848","Cervid1","D","ETH152","INRA011","K","N","O","OarFCB193","Q"))

#above includes the inputted missing values, replace to create the input file for RMES

write.table(ifelse(genotypes.weight.complete==0,0,ifelse(genotypes.weight.complete==1,1,-99)),"MASSgenotypes.txt",sep=" ",col.names=F,row.names=F)

#again, the first lines of this file need to be modified, see above and RMES instructions

#RMES produces

#g2=0.013

#other stats

mean(rowSums(genotypes.weight.complete)) #E(H) = 9.333

var(rowSums(genotypes.weight.complete)) #var(H) = 2.719

# r.H.f = ( g2*E(H)^2 ) / var(H) =

(0.013*9.333^2)/2.719 #0.42

0.0165/0.42 #r.f.T

####################################################################################################

# analysis of the other traits is performed similarly as illustrated for body mass

####################################################################################################

#exploration of local effects

####################################################################################################

wtd.weight.genotypes<-wtd.dat[complete.cases(subset(dryad.wtd.dat,select=c("weight","age.class","Sex","Sum.H"))),]

mod.gen<-(lm(formula = weight ~ -1 + age.class + Sex + Sum.H + age.class:Sex,data = wtd.weight.genotypes))

mod.loc<-(lm(formula = weight ~ -1 + age.class + Sex + BL25 + BM203 + BM6438 + BM6506 + BM848 + Cervid1 + D + ETH152 + INRA011 + K + N

+ O + OarFCB193 + Q + age.class:Sex,data = wtd.weight.genotypes))

anova(mod.gen,mod.loc) #test for equality of fit for competing models

######################################################################

#¤¤¤¤¤¤¤¤¤ simulation for power when reducing number of msats

######################################################################

rounds=10000

min.n.msats=5

P.mat=matrix(NA,rounds,(13-min.n.msats+1))

for (m in min.n.msats:13) {

for (r in 1:rounds) {

#select random set of msats

#msats are cols 13:26

wtd.sim<-wtd.dat[,1:12]

wtd.sim$Sum.H.sim<-rowSums(wtd.dat[,(12+sample(14,m))])

wtd.weight.complete.sim<-subset(wtd.sim,select=c("weight","age.class","Sex","Sum.H.sim"))

wtd.weight.complete.sim<-wtd.weight.complete.sim[complete.cases(wtd.weight.complete.sim),]

P.mat[r,(m-(min.n.msats-1))]<-summary(lm(formula = weight ~ -1 + age.class + Sex + Sum.H.sim + age.class:Sex,data = wtd.weight.complete.sim))$coeff[7,4]

}

}

power<-colSums(P.mat<0.05)/rounds

plot(power)
